# *Enterococcus faecalis* Endocarditis Severity in Rabbits Is Reduced by IgG Fabs Interfering with Aggregation Substance

**DOI:** 10.1371/journal.pone.0013194

**Published:** 2010-10-04

**Authors:** Patrick M. Schlievert, Olivia N. Chuang-Smith, Marnie L. Peterson, Laura C. C. Cook, Gary M. Dunny

**Affiliations:** Department of Microbiology, University of Minnesota Medical School, Minneapolis, Minnesota, United States of America; National Institute of Allergy and Infectious Diseases, National Institutes of Health, United States of America

## Abstract

**Background:**

*Enterococcus faecalis* is a significant cause of infective endocarditis, an infection of the heart endothelium leading to vegetation formation (microbes, fibrin, platelets, and host cells attached to underlying endothelial tissue). Our previous research determined that enterococcal aggregation substance (AS) is an important virulence factor in causation of endocarditis, although endocarditis may occur in the absence of AS production. Production of AS by *E. faecalis* causes the organism to form aggregates through AS binding to enterococcal binding substance. In this study, we assessed the ability of IgGs and IgG Fabs against AS to provide protection against AS^+^ *E. faecalis* endocarditis.

**Methodology/Principal Findings:**

When challenged with AS^+^ *E. faecalis*, 10 rabbits actively immunized against AS^+^ *E. faecalis* developed more significant vegetations than 9 animals immunized against AS^−^ *E. faecalis*, and 9/10 succumbed compared to 2/9 (p<0.005), suggesting enhanced aggregation by IgG contributes significantly to disease. IgG antibodies against AS also enhanced enterococcal aggregation as tested in vitro. In contrast, Fab fragments of IgG from rabbits immunized against purified AS, when passively administered to rabbits (6/group) immediately before challenge with AS^+^ *E. faecalis*, reduced total vegetation (endocarditis lesion) microbial counts (7.9×10^6^ versus 2.0×10^5^, p = 0.02) and size (40 mg versus 10, p = 0.05). In vitro, the Fabs prevented enterococcal aggregation.

**Conclusions/Significance:**

The data confirm the role of AS in infective endocarditis formation and suggest that use of Fabs against AS will provide partial protection from AS^+^ *E. faecalis* illness.

## Introduction

*Enterococcus faecalis* is a significant cause of infective endocarditis, an infection of the endothelium of the heart [1], [2]. The organism has been known to cause endocarditis since the early 1900s, and is now considered one of the most significant causes of endocarditis, resulting in as many as 10–15% of cases [3], [4]. The characteristic endocarditis lesions are referred to as vegetations, which appear as nodules containing microbes, fibrin, platelets, and host cells attached to underlying endothelial tissue [3].

Previous studies have determined that enterococcal aggregation substance (AS), although not required for enterococci to produce endocarditis, when present contributes significantly to the ability of *E. faecalis* to cause endocarditis [5], [6], [7]. AS is a large (137 kDa) surface-exposed protein encoded by pheromone-responsive, conjugative plasmids. This protein contributes to formation of large bacterial cell aggregates through binding to enterococcal binding substance (EBS), a component of which appears to include lipoteichoic acid [8]. The AS proteins comprise a family of surface adhesins whose amino acid sequences are >90% identical through most of the proteins, with exception of a small internal segment of the protein that is more variable; our laboratories study Asc10, an AS protein encoded by the plasmid pCF10 [9], [10]. Through use of isogenic strains, we have demonstrated that AS^+^ *E. faecalis* cause larger endocarditis vegetations with higher bacterial loads than AS^−^ organisms in a rabbit endocarditis model. The cumulative results of analysis of Asc10 suggest that the protein has at least two different functional activities in endocarditis pathogenesis, probably associated with different domains [8], [10], [11], [12]. An N-terminal domain confers bacterial aggregation and lipoteichoic acid binding activity, and also plays a role in interactions of enterococci with mammalian host cells [8]. In addition, the protein contains two arginine-glycine-aspartic acid (RGD) motifs potentially mediating interactions with integrins, and there is suggestive evidence that these domains might be involved in protection of enterococcal cells from killing by phagocytes [8], [12].

In spite of the abundance of data for a contribution of AS to the pathogenesis of enterococcal endocarditis, our studies have shown that antibodies raised against a surface-exposed, N-terminal domain of AS are not protective against endocarditis in a rabbit model [6], [13]. Multiple hypotheses have been presented to account for lack of antibody protection. One of these states that antibodies to AS may actually enhance virulence due to ability to promote additional aggregation of enterococcal cells. This hypothesis was evaluated in the present studies in which we assessed the ability of IgG Fab fragments against AS to provide protection against AS^+^ *E. faecalis* endocarditis.

## Results

**Figure 1:**
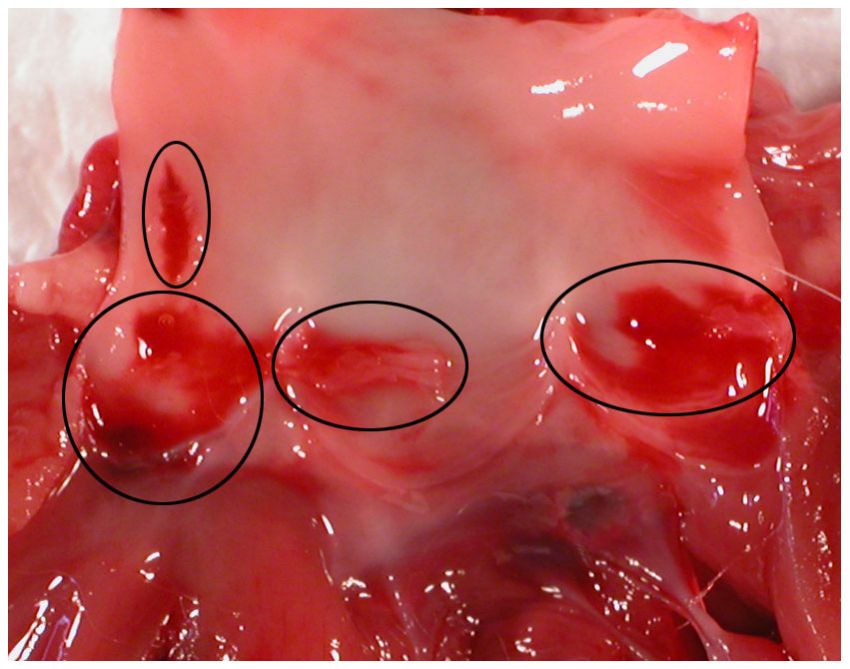
*Enterococcus faecalis* OG1SSp (pINY1801, AS^+^) infectious endocarditis aortic valve and aortic vegetations. Vegetations are circled.

Rabbits (3/group) were injected intravenously with AS^+^ *E. faecalis* strain OG1SSp carrying the plasmid pINY1801, expressing the cloned AS gene (*prgB*) constitutively, or the isogenic AS^−^ strain OG1SSp carrying the same cloning vector plasmid (referred to as pWM401) without the AS gene. The bacterial strains were administered after 2 h of continuous damage to the aortic valves/aortas by prior catheterization. After 4 additional days, the rabbits were sacrificed and examined for vegetations. All animals had visible vegetations, but those obtained from rabbits inoculated with the AS^+^ strain were larger and had higher bacterial loads than animals receiving the AS^−^ strain (Table 1), in agreement with previous results [5], [6], [7], [14]. An example of a rabbit aorta with vegetations induced by the AS^+^ strain is shown in Fig. 1 (vegetations are circled).

**Table 1:** Enterococcal aggregation substance enhances vegetation production in endocarditis in rabbits.

Treatment Group	Number of Rabbits	Mean Vegetation Weight (mg)	*P*-Value	Mean Log CFU Total in Vegetations	*P*-Value
OG1SSp (AS^+^)	3	62		7.7	
OG1SSp (AS^−^)	3	39	0.04	5.6	0.03

Rabbits (9 or 10 per group) were immunized against either *E. faecalis* OG1SSp (pINY1801, AS^+^) or isogenic *E. faecalis* OG1SSp (pWM401, AS^−^). The animals were then challenged with OG1SSp (pINY1801, AS^+^) to assess immunization against AS to protect rabbits from infective endocarditis. The rabbits that had been immunized with the AS^+^ organism showed more significant vegetations than those immunized against the AS^−^ organism or those animals that were not immunized (Table 2). In addition, animals immunized with the AS^+^ organism showed extensive lung congestion, and all but one of the animals succumbed prior to the end of the study; the 9 rabbits succumbed during day 2 of experimentation. Collectively, these animals had vegetations of sufficient size and numbers to obstruct their aortas. In contrast, only 2 of the 9 animals immunized with the AS^−^ organism succumbed (also during day 2) when challenged with the AS^+^ organism, and none succumbed in the non-immunized group. Animals that had been immunized with the AS^−^ organism had signs of lung congestion, but not to the extent of animals immunized against the AS^+^ organism and challenged with AS^+^ organism. The vegetations in the animals immunized with the AS^−^ organism, or those that were not immunized had moderate size and numbers of vegetations.

**Table 2:** Active immunization against AS^+^ *E. faecalis* worsens infectious endocarditis after challenge with AS^+^ enterococci.

Rabbits Immunized Against:	Alive/Total Tested	*P*-Value Compared to AS^+^ *E. faecalis* Immunized Animalsa−	Vegetationsb−	Respiratory Condition
AS^+^ *E. faecalis*	1/10		3+	pneumonia
None	5/5	0.002	2+	Normal
AS^−^ *E. faecalis*	7/9	0.005	2+	pneumonia

^a^−Determined with use of Fishers Exact Test.

^b^−1+ vegetations were small in size and number (approximately 10 mg); 2+ vegetations were moderate in size and number (approximately 50 mg); and 3+ vegetations were large in size and numbers, often obstructing the aorta (>75 mg).

It was hypothesized previously that antibodies raised against the N-terminus of AS may enhance ability to cause endocarditis [6]. The above experiment in which rabbits were actively immunized with killed OG1SSp (pINY1801, AS^+^) organisms and then challenged with viable OG1SSp (pINY1801, AS^+^) also suggested that antibodies to AS enhance disease. In order to test this hypothesis more fully and to identify a potential therapeutic agent, Fabs of hyperimmune IgG raised against purified AS were prepared, confirmed by SDS-PAGE, and used passively in attempt to prevent OG1SSp (pINY1801, AS^+^) ability to cause endocarditis (Table 3). The mean weight of vegetations of the rabbits treated passively with Fabs was 10 mg, compared to 40 mg for animals not receiving passive Fabs (*p*<0.05). There was a greater than two-log difference in total log CFUs of enterococci in the vegetations from the Fab treated rabbits compared to control animals (*p*<0.02). There was no evidence of pulmonary congestion in any of the Fab treated rabbits, but mild congestion was observed in two of the 6 control animals; none of the 12 animals used in this study prematurely succumbed.

**Table 3:** IgG Fabs against aggregation substance reduce the severity of enterococcal endocarditis after challenge with AS^+^ *E. faecalis*.

Treatment Group	Number of Rabbits	Mean Vegetation Weight (mg)	*P*-Value	Mean Log CFU Total in Vegetations	*P*-Value
Control	6	40		6.9	
Fab Treated	6	10	0.05	4.3	0.02

**Figure 2:**
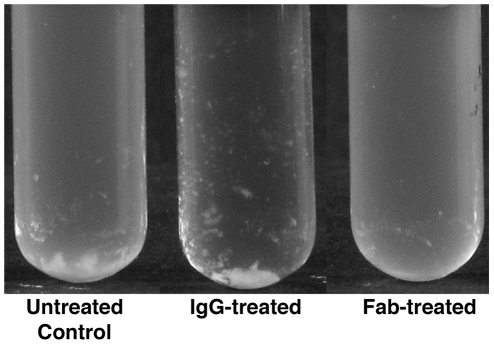
IgG antibodies to enterococcal aggregation substance enhance aggregation of *E. faecalis* in vitro, whereas Fabs prevent aggregation.

The ability of IgG against AS to enhance the aggregation of AS^+^ *E. faecalis* OG1SSp (pINY1801) in vitro and Fabs to inhibit aggregation were assessed visually (Fig. 2). As expected, AS^+^ *E. faecalis* OG1SSp (pINY1801) spontaneously aggregated forming moderate aggregates of bacteria at the bottom of the tube, but with some bacteria remaining in the culture supernate. The aggregation of AS^+^ *E. faecalis* OG1SSp (pINY1801) was enhanced by IgG antibodies to AS in that the aggregates of bacteria at the bottom of the tube were larger, formed more quickly, and nearly completely cleared the culture supernate. Fabs against AS completely prevented visible aggregation of AS^+^ *E. faecalis* OG1SSp (pINY1801).

## Discussion

Previous research by our group [5], [6], [8], [13], [14] and by other investigators [15] suggests that enterococcal AS is an important virulence factor in causation of infective endocarditis. However, the factor is not required for inducing endocarditis, as AS^−^ organisms also have the ability to induce illness [5], [14]. We previously showed that isogenic AS^−^ organisms cause more inflammation pericardially compared to AS^+^ organisms when both organisms are injected into the hearts of rabbits [5], [14]. These data suggest there are different pathways that lead to vegetation formation, one in which there is significant activation of the host immune system to clear the organism, as with the AS^−^ organism, and one in which larger vegetations form in the absence of high-level immune system activation, possibly due to formation of aggregates, as with the AS^+^ organism.

Having established that AS contributes to the virulence of *E. faecalis* through our studies and those of others, we attempted to interfere with AS activity, and thus, possibly reduce the severity of endocarditis. One observable phenotypic function of AS is aggregate formation through interaction with EBS that includes lipoteichoic acid [8]. It could then be hypothesized that further aggregate formation through reaction of IgG antibodies with AS, with or without complement fixation, might increase the severity of endocarditis. This was observed in our studies. In addition, the formation of very large aggregates would be expected to obstruct small capillaries, such as those in the lungs, leading to pneumonia-like symptoms. This was also observed in rabbits immunized with both AS^+^ and AS^−^ organisms and challenged with AS^+^ organisms (the lung congestion was greater in animals immunized with the AS^+^ organisms). With this as background, we evaluated the ability of Fabs of IgG antibodies raised specifically against AS to interfere with endocarditis. We studied Fabs of IgG rather than F(ab')_2_ molecules since Fabs lack ability to both cross-link antigens and fix complement, whereas F(ab')_2_ molecules retain ability to cross-link antigens, though they have lost ability to fix complement. We first raised IgG antibodies against a secreted form of intact AS [8], then partially purified the IgGs and made Fabs. These Fabs, when passively administered intravenously to rabbits immediately before challenge with AS^+^ organisms, reduced both vegetation size and CFUs of enterococci in vegetations. The in vivo effects of IgG and Fabs against AS were confirmed by in vitro tests in which IgGs enhanced enterococcal aggregation and Fabs completely interfered with aggregation.

The studies are significant for two reasons: 1) the ability of AS to induce aggregation is important in endocarditis formation, although endocarditis may occur in their absence (as observed with the AS^−^ organisms); and 2) use of passive Fabs against surface microbial virulence factors in general may be more effective than IgGs against the same virulence factors. Thus, for example, other organisms that cause significant endocarditis include *S. aureus* and viridans streptococci. These organisms have many cell-surface virulence factors referred to as microbial surface components recognizing adhesive matrix molecules (MSCRAMMs), at least some of which lead to aggregate formation [16]. Attempts are underway to develop antibody-based vaccines against the organisms by targeting surface virulence factors, including MSCRAMMs [17]. It is possible that such antibodies would be more effective if administered as Fabs that prevent aggregate formation.

## Materials and Methods

### Plasmids and Bacteria

The plasmid pWM401 is a broad-host range cloning vector; pINY1801 contains a cloned fragment of pCF10, including the Asc10-encoding *prgB* gene [18]. It has been previously shown that *E. faecalis* strains carrying pINY1801 produce AS constitutively (5, 14). Isogenic *E. faecalis* strains OG1SSp (pINY1801) and OG1SSp (pWM401, differing only in production of AS, were used as the test organisms for immunization and production of endocarditis [5]. The organisms were cultured overnight in a dialyzable beef heart medium [19], washed one time with PBS, and resuspended to 1×10^9^ CFU/ml in PBS; 2 ml were injected intravenously into the marginal ear veins of rabbits for endocarditis production [5]. OG1SSp (pINY1801) was verified as AS^+^ by its ability to form visible aggregates during growth; OG1SSp (pWM401) lacked the ability to form aggregates. Bacterial strains were stored as lyophilized stock cultures in the laboratory until used.

### Antiserum and Fabs

Hyperimmune antiserum against AS was prepared by immunization of rabbits against purified AS [8] emulsified in Freund's incomplete adjuvant (Sigma Aldrich, St. Louis, MO). This serum was determined experimentally to have a titer (reciprocal of last reactive wells in 96 well microtiter plates) against AS of 2500/ml, as determined by ELISA. The IgG fraction of this antibody was prepared by ammonium sulfate precipitation (final ammonium sulfate concentration 34%) and resolubilization in PBS. The collected IgG fraction was dialyzed for two days against PBS to remove residual ammonium sulfate, and the resultant IgG fraction was digested to completion with papain to generate Fabs. Complete digestion was verified by SDS-PAGE in non-reducing gels [20] as demonstrated through loss of the IgG fraction and appearance of a strong band with a molecular weight of approximately 50,000.

Each rabbit receiving Fab fragments was injected intravenously with the equivalent of approximately 2.5 ml of original hyperimmune antiserum. Assuming the injected rabbits had blood volumes of approximately 50 ml, each rabbit received sufficient Fabs to achieve titers of 125/ml blood.

In one in vitro experiment, 0.5 ml of ammonium sulfate fractionated IgG against AS or 0.5 ml of Fabs against AS were mixed with 0.5 ml volumes of stationary phase cultures of AS^+^ *E. faecalis* OG1SSp (pINY1801) for 30 min at room temperature and then examined visually for degree of aggregation.

### Immunization Against Enterococci

*E. faecalis* OG1SSp (pINY1801, AS^+^) and OG1SSp (pWM401, AS^−^) were cultured to stationary phase in beef heart medium. The organisms were washed one time with PBS and adjusted to 2×10^9^ CFU/ml in PBS and killed by heat (65°C, 30 min) and then gentamicin (100 ug/ml, 1 h, 37°C). The cells (0.5 ml amounts) were emulsified with equivalent amounts of Freund's incomplete adjuvant and administered subcutaneously in the nape of the neck to rabbits (9 or 10 per test organism). The animals were injected 3 times separated by two week intervals and then used for challenge with AS^+^ *E. faecalis* OG1SSp (pINY1801) to assess the ability of active immunization to provide protection against infective endocarditis. Prior to challenge, the rabbits were evaluated for antibody titers against the corresponding organisms with use of ELISA. Wells of Nunc-Immuno plates were coated with 100 ul/well of 2×10^9^ CFU/ml of the immunizing enterococci for use in antibody titer determination. Average antibody titers to the immunizing enterococci, whether AS^+^ *E. faecalis* OG1SSp (pINY1801) or AS^−^ *E. faecalis* OG1SSp (pWM401), were >10,000, compared to ≤10 for preimmune sera.

### Experimental Endocarditis

All animal studies were performed in accordance with requirements established by the University of Minnesota Institutional Animal Care and Use Committee, with specific approval given for this work under protocol 0910A73332 (project name Biofilms and *Enterococcus faecalis* Biology). Healthy New Zealand white rabbits, either sex and weighing approximately 2 kg, were obtained from Bakkom Rabbitry, Red Wing, MN. After acclimating to the University of Minnesota animal facilities for approximately one week, the rabbits as the model animal for infective endocarditis [5] were challenged with *E. faecalis*. Briefly, rabbits were anesthetized with ketamine and xylazine for a total of approximately 4 h. The left carotid artery of each animal was surgically exposed, a catheter (outside diameter 1.27 mm) was inserted into the carotid to cause damage to the aortic valve for 2 h, each catheter was then removed, each carotid was ligated, the neck incision was closed, and each animal was challenged intravenously with viable organisms through the left marginal ear vein. Animals were sacrificed on day 4 with a lethal injection of Beuthanasia D, and hearts were resected. Vegetations were exposed, photographed, aseptically removed, weighed, and homogenized in PBS for quantitative bacterial counts.
